# Polish psychiatrists’ experiences consulting displaced patients from ukraine in 2022

**DOI:** 10.1192/j.eurpsy.2024.370

**Published:** 2024-08-27

**Authors:** K. Krysta, K. Kamińska, Z. Mielczarek, M. Ciołek

**Affiliations:** Department of Rehabilitation Psychiatry, Medical University of Silesia, Katowice, Poland

## Abstract

**Introduction:**

The ongoing conflict in Ukraine has resulted in a significant influx of refugees seeking asylum in other countries, including Poland. Among these refugees are individuals who are struggling with mental health issues. Polish psychiatrists have stepped up to provide care for these patients, despite facing a number of challenges in the process.

**Objectives:**

This presentation aims to shed light on the experiences of Polish psychiatrists treating refugees during the war in Ukraine, highlighting the difficulties they have encountered and the strategies they have employed to provide the best possible care to their patients. The presentation also examines the impact of war on mental health, and the long-term effects on the well-being of refugees.

**Methods:**

A questionnaire study was done among Polish Psychiatrists about the forms of support they provided for Ukrainin psychiatric patients they consulted after 24 February 2022. The responses to questionnaires were collected during psychiatric Congresses.

**Results:**

The most commonly reported symptoms were anxiety (44.1%), followed by depression (35.3%), and panic attacks (23.5%). Other symptoms like irritability and sleep disorders were reported by 11.8% and 8.8% of the respondents, respectively. Disturbingly, thoughts of resignation and suicidal ideation were also reported, albeit at lower frequencies (8.8% and 2.9%, respectively). A small percentage (2.9%) reported no new symptoms.

The high prevalence of anxiety and depression suggests that the war has had a profound impact on the mental health of the affected population. The emergence of severe symptoms like psychotic thoughts and suicidal ideation, although less frequent, is alarming and calls for immediate intervention. It is also noteworthy that a small but significant portion of the population reported no new symptoms, which may indicate resilience or other coping mechanisms at play.

**Conclusions:**

The war in Ukraine has led to a range of new psychological symptoms among the affected populations, with anxiety, depression, and panic attacks being the most prevalent. Immediate and long-term psychological interventions are urgently needed to address these emerging mental health issues. Further research is also required to understand the resilience factors among those who reported no new symptoms.

**Disclosure of Interest:**

None Declared

